# Genome-Wide Identification of Human Functional DNA Using a Neutral Indel Model

**DOI:** 10.1371/journal.pcbi.0020005

**Published:** 2006-01-13

**Authors:** Gerton Lunter, Chris P Ponting, Jotun Hein

**Affiliations:** 1 MRC Functional Genetics Unit, Department of Human Anatomy and Genetics, University of Oxford, Oxford, United Kingdom; 2 Department of Statistics, Bioinformatics Group, University of Oxford, Oxford, United Kingdom; Fred Hutchinson Cancer Research Center, United States of America

## Abstract

It has become clear that a large proportion of functional DNA in the human genome does not code for protein. Identification of this non-coding functional sequence using comparative approaches is proving difficult and has previously been thought to require deep sequencing of multiple vertebrates. Here we introduce a new model and comparative method that, instead of nucleotide substitutions, uses the evolutionary imprint of insertions and deletions (indels) to infer the past consequences of selection. The model predicts the distribution of indels under neutrality, and shows an excellent fit to human–mouse ancestral repeat data. Across the genome, many unusually long ungapped regions are detected that are unaccounted for by the neutral model, and which we predict to be highly enriched in functional DNA that has been subject to purifying selection with respect to indels. We use the model to determine the proportion under indel-purifying selection to be between 2.56% and 3.25% of human euchromatin. Since annotated protein-coding genes comprise only 1.2% of euchromatin, these results lend further weight to the proposition that more than half the functional complement of the human genome is non-protein-coding. The method is surprisingly powerful at identifying selected sequence using only two or three mammalian genomes. Applying the method to the human, mouse, and dog genomes, we identify 90 Mb of human sequence under indel-purifying selection, at a predicted 10% false-discovery rate and 75% sensitivity. As expected, most of the identified sequence represents unannotated material, while the recovered proportions of known protein-coding and microRNA genes closely match the predicted sensitivity of the method. The method's high sensitivity to functional sequence such as microRNAs suggest that as yet unannotated microRNA genes are enriched among the sequences identified. Futhermore, its independence of substitutions allowed us to identify sequence that has been subject to heterogeneous selection, that is, sequence subject to both positive selection with respect to substitutions and purifying selection with respect to indels. The ability to identify elements under heterogeneous selection enables, for the first time, the genome-wide investigation of positive selection on functional elements other than protein-coding genes.

## Synopsis

Despite the major impact of sequencing the human genome on our understanding of biology, a fundamental problem remains. Many of the genome's functional elements, particularly those that do not encode protein, are proving difficult to distinguish from neutrally evolving DNA. Lunter et al. introduce a method that exploits the evolutionary imprint of sequence insertions and deletions (so-called indels) to pinpoint functional DNA regions that have been subject to purifying selection. This method hinges on a simple theoretical prediction for the distribution of indels across the human genome. Despite its simplicity, the model shows an excellent fit to human and mouse alignments. This tight fit has been exploited to show that virtually all ancient transposable elements are evolving neutrally, which has long been suspected but not quantified. Indeed, the model estimates the probability that, among all alignable human sequence, a region has been purged of deleterious indels since the human–mouse split. This leads to the prediction that between 2.56% and 3.25% of the human genome sequence is functional. Importantly, the method is independent of conventional nucleotide substitution approaches, and thus immediately presents an initial opportunity to investigate the impact of positive selection on non-coding functional elements.

## Introduction

The human genome has been shaped by the evolutionary forces of mutation, genetic drift, and selection, with the latter acting, in the main, to purify functional regions of deleterious mutations. By comparing the human and mouse genomes, previously it was estimated that about 5% of the human genome has undergone fewer point mutations than expected under a neutral substitution model [1,2]. Accepting that this is most likely caused by the effects of purifying selection acting on deleterious mutations, the observation implies that at least 5% of the human genome is biologically functional. Since the only known large class of functional genomic elements, protein-coding exons, is believed to constitute only 1.2% of our genome [3], this remains a surprising result.

To begin to understand the biological role of the remaining non-genic functional elements, the essential first step is their identification. Recent studies have focussed on the most highly conserved of these elements, namely ultraconserved elements (defined as segments of >200 base pairs [bp] without substitutions) [4]. These elements exhibit a surprisingly high level of conservation that is rare even among protein-coding exons, and studies have begun to suggest intriguing roles of such elements in alternative splicing and development [5,6]. However, the vast majority of non-genic elements are not perfectly conserved with respect to point mutations, and the reliable identification of these elements within a sea of neutrally evolving DNA has proved difficult. Deep conservation among diverse phyla is a reliable sign of conserved biological function, but is less suitable for identifying recently evolved sequence. Comparative methods for closely related species typically analyze substitution patterns to flag conserved regions [7]. These methods are well-developed, and they exploit phylogenetic information and correlations along the sequence to achieve high sensitivities. Although extremely powerful, these methods can be hard to calibrate because of incompletely understood variations in neutral rates of substitution due to, for instance, methylation levels, chromatin state, transcriptional activity, and chromosomal location, and conservative calibration leads to a reduction of sensitivity. Deep sequencing of mammalian genomes considerably improves the power of comparative methods [8], and while expensive, this will eventually represent the most satisfying solution to the sensitivity problem.

Of all mutation processes, point substitutions are the most prevalent, with insertions and deletions (indels) approximately 10-fold less frequent. While nucleotide substitution models have been studied intensively [9,10], with the exception of gene finding [11] indels have largely been treated as evolutionary “nuisance events,” to be accounted for by alignment procedures, but otherwise uninformative. Contrary to this view, we show that indels are highly informative evolutionary events. We introduce a model describing the neutral distribution of indels over the genome, and show that this model fits a large proportion of human–mouse alignment data remarkably well. We then show that deviations from the model are, in the main, not caused by variations in the neutral indel rates, but are consistent with selection acting to purify the genome of deleterious indels that arise in functional regions.

We first applied this neutral indel model to derive upper and lower bounds on the proportion of genome under purifying selection with respect to indels (indel-purifying selection). Our observations can be explained by proposing that between 78.8 ± 0.6 Mb and 100.0 ± 0.8 Mb (2.56%–3.25%) of the human genome has been under indel-purifying selection since the human–mouse split. Although still much higher than the 1.2% represented by coding exons, this represents a substantially lower estimate than the previous 5% estimate based on substitution-level conservation [1,2], but is consistent with a more recent estimate [7]. Restricting ourselves to ancestral repeats (ARs), transposable elements (TEs) inserted before the human–mouse split, we found a near-exact fit between observations and the neutral model predictions. Applying the same method as before, we predict that among the 1,263 Mb of TEs, only at most 1.2 Mb (0.09%) have been under sustained purifying selection. This is the first time to our knowledge that a model of neutral evolution has quantified the proportion of TEs that have evolved neutrally.

As a second application of the neutral indel model, we identified a large proportion of sequence elements that have evolved under indel-purifying selection. The model allowed us to calculate the predicted false-discovery rate (FDR) for the entire set, as well as Bayesian posterior probabilities for individual elements to be under indel-purifying selection. By correlating this set with various independent functional indicators, both positive (for example, overlap with, or close proximity to, known exons) and negative (TE annotation), it is shown to be highly enriched with functional DNA.

The key strength of the proposed method lies in its independence of selection with respect to point mutations. Consequently, the method can provide independent confirmation of selection, thereby improving the specificity of methods based on substitutions alone. Moreover, an exciting possibility is that the method allows identification of sequence elements that have been under heterogeneous selection, i.e., that have been subject to purifying selection with respect to indels, but subject to positive selection or relaxed constraints with respect to substitutions. Examples of such elements would include spacers between regulatory elements whose relative distance is functionally constrained, such as those shown to exist in *Drosophila* [12]. Although functional, the nucleotide sequence of such spacers is probably immaterial, implying relaxed constraints with respect to substitutions. An even more interesting class consists of elements whose sequence is under positive selection with respect to substitutions, while at the same time under purifying selection with respect to indels. Since indels can be highly disruptive of function in protein-coding and RNA genes, as is evident from the 10-fold reduced indel rates in exons compared with neutrally evolving DNA, it is conceivable that such elements exist. Without exploiting the evolutionary imprint of indel-purifying selection, it is difficult to see how to identify functional elements under positive selection with respect to substitutions in the absence of a comprehensive functional annotation, which only exists currently for protein-coding genes. An analysis of percent sequence identity suggested that as much as 5% of DNA under indel-purifying selection, or roughly 3–5 Mb, may be under heterogeneous selection. Among the indel-conserved elements identified, those that exhibit more than the expected number of substitutions for neutrally evolving DNA still showed correlations with the functional indicators mentioned above, thereby further confirming the existence of elements under heterogeneous selection.

## Results

### The Neutral Indel Model

The neutral indel model hinges on two assumptions: that distinct indel events are independent, and that they occur uniformly across the genome. The first assumption likely holds to high accuracy, but indel rate uniformity can only be expected to be approximately valid; we thus eventually account for indel rate variation in the later analysis (see the section Accounting for Indel Rate Variation). However, accepting both assumptions as a first approximation, we can immediately draw the conclusion that the distance between successive indels, measured as the number of homologous nucleotides surviving in between, follows a geometric distribution. Note that this conclusion holds irrespective of the distribution of indel lengths themselves, and of the relative incidence of insertions and deletions.

The fact that indel events often involve several nucleotides simultaneously introduces co-dependencies in the survival probabilities of nearby sites. In other words, the probability that an ancestral nucleotide survives as a homologous nucleotide in two descendant species is dependent on whether neighbouring nucleotides survive. However, assuming independence of indel events, survival probabilities do become independent conditional on the survival of the left (or right) neighbour. Indeed, if *p* is the uniform conditional survival probability for a single nucleotide, the (conditional) survival probability of a sequence of *L* nucleotides is *p^L^* because of the assumption of independence. In this paper, we refer to *ρ =* 1*−p* as the indel probability per site, or less precisely, the indel rate.

**Figure 1:**
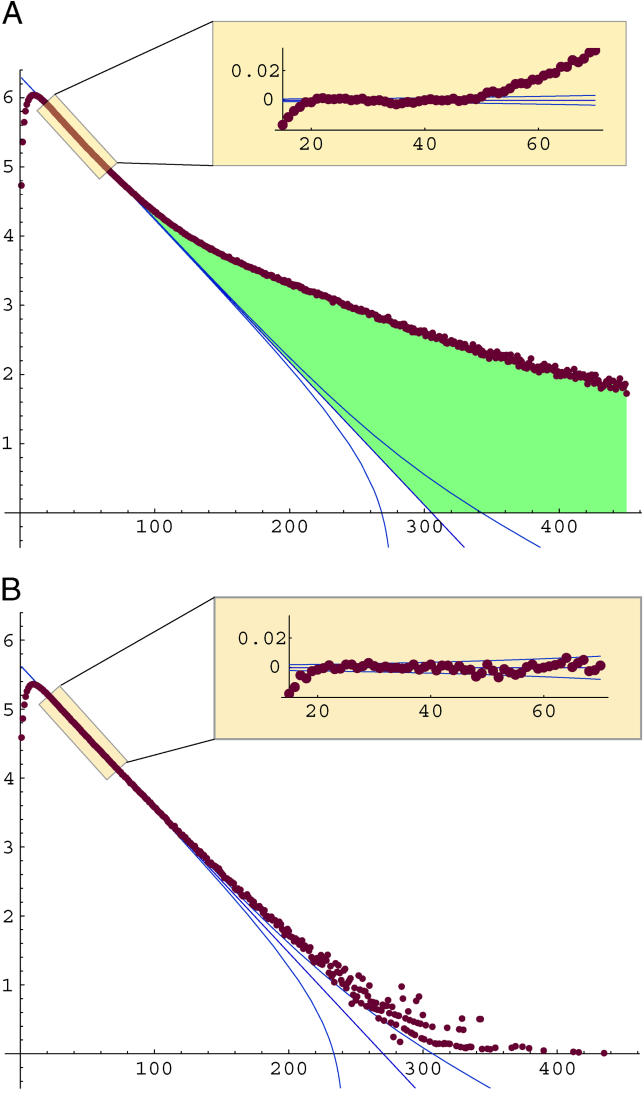
Genomic Distribution of Intergap Distances Histogram of intergap distance counts (log_10_ scale) in human–mouse alignments, (A) within the whole genome and (B) within ARs. Blue lines indicate predictions of the neutral model (central line, geometric distribution; the slope is related to the per-site indel probability *ρ*), and expected sampling errors (outer curves; 95% confidence intervals for a binomial distribution per length bin). Insets show a blow-up of the deviation from the model (log_10_ scale). Parameters were obtained by linear regression to the log-counts, weighted by the expected binomial sampling error. The indel distribution on AR data shows an excellent model fit, in particular in the range 20–80 bp, with 92% of counts (56/61) lying within 95% confidence limits. The whole-genome histogram shows a similarly tight fit in the range 20–50 bp, and a large excess of long intergap distances over neutral model predictions (green) beyond ~50 bp. The intercept of the geometric prediction occurs at a length *L* = 300. This implies that less than one ungapped sequence of any length *L* > 300 is expected genome-wide under the neutral model; however the model does predict a small but nonzero probability for any such sequence, even under neutrality.

Although indels cannot be observed directly, for the low indel rates observed in mammals they closely correspond to gaps in the alignment. It thus may be predicted that, under neutrality, the lengths of ungapped sequence between successive alignment gaps—intergap segments (IGS)—would be distributed similarly to the geometric distribution predicted for the distance between successive indels. A whole-genome histogram of IGS lengths, obtained from BlastZ human–mouse alignments [13] indeed shows a remarkably close fit to the geometric distribution (a straight line in log-linear coordinates) within the length range 20–50 bp, with the model explaining 99.996% of the variance (Figure 1A). To show that this close fit is not caused by alignment artefacts, human Chromosome 21 was realigned to orthologous sequence in mouse using a simple probabilistic aligner and three sets of parameters. The resulting histograms show similarly close fits within the range 20–50 bp, with the *ρ* parameters within 95% confidence intervals of one another (see Materials and Methods).

Outside of the range of 20–50 bp, histogram counts deviate from the neutral model predictions, with IGS of less than 20 bp being underrepresented, and IGS longer than 50 bp being overrepresented. The underrepresentation of short intergap distances is caused by a systematic alignment artefact termed gap attraction [14], by which two nearby indel events give rise to a single alignment gap when this more parsimoniously explains the observed sequence data. This phenomenon does not reflect an evolutionary process, and thus, in what follows, ungapped segments shorter than 20 bp were ignored.

To investigate whether the overrepresentation of long ungapped segments is, to a large extent, caused by indel-purifying selection, a similar histogram was constructed using only alignments of ARs (see Materials and Methods). These elements are thought to evolve predominantly neutrally [15,16], and the histogram obtained indeed closely followed the predictions of the neutral model, with only a slight overrepresentation of long ungapped segments (Figure 1B). These observations are further quantified below.

### Accounting for Indel Rate Variation

To quantify the extent of any deviation of the intergap histogram from the neutral model, we introduced a parameter *σ*. This parameter measures the fraction of nucleotides in ungapped segments that are overrepresented in the genome (or among ARs) compared to the prediction of the neutral model (see Materials and Methods), and is visually represented in green in Figure 1A. For the whole-genome and AR histograms, *σ* was determined to be 0.1234 and 0.0074, respectively. (Note that *σ* is not an estimate of the proportion of nucleotides under selection, although it does provide a rough upper bound. More precise bounds for this proportion are derived below.)

Generally, a local reduction of the effective indel rate (i.e., the indel rate resulting from mutation and selection combined) gives rise to an overrepresentation of long ungapped segments, as measured by *σ*. Although purifying selection, which causes strongly reduced rates over considerable lengths, for instance in protein-coding exons, contributes greatly to *σ,* a variation in neutral rates would also. Neutral indel rate variation would cause the IGS-length distributions under neutrality to be a mixture of geometrics. Such a mixture distribution is convex in log-linear coordinates, and a fit to a simple geometric would result in a positive *σ* value.

**Figure 2:**
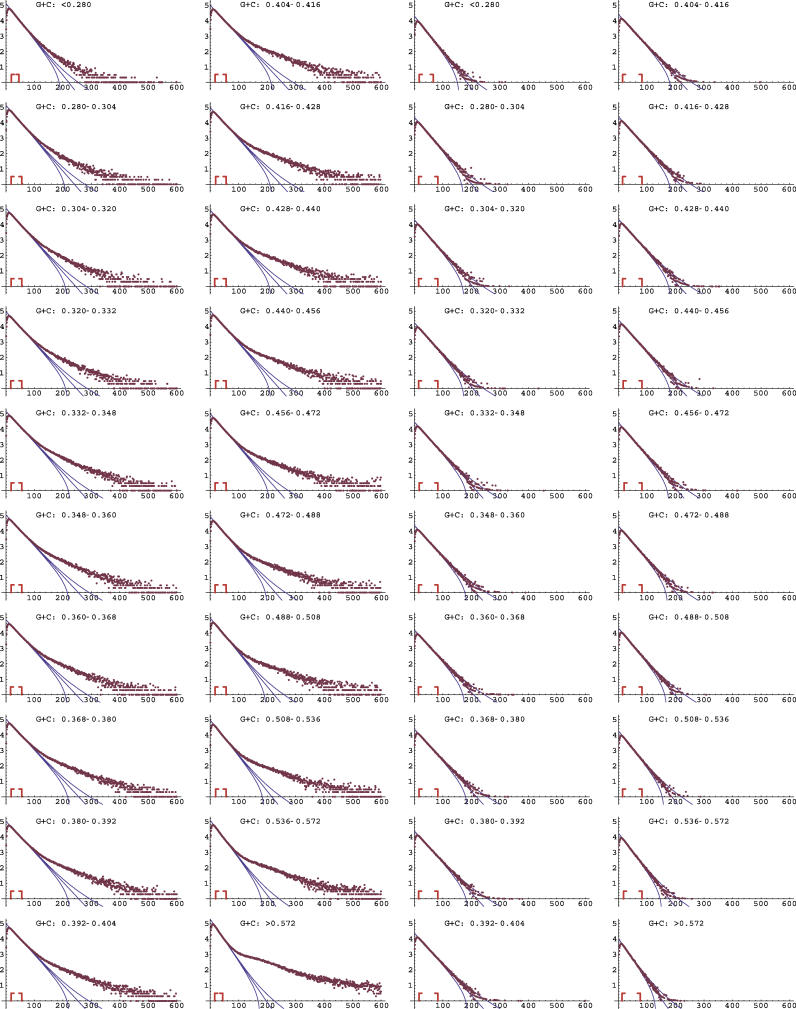
Intergap Distance Distribution by G+C Content Intergap distance histograms, per G+C content bin, for all of the autosomes and the Y chromosome (Left hand columns) and restricted to ARs within these chromosomes (Right hand columns). Horizontal axes, inter-gap distance (nucleotides); vertical axes, log_10_ counts. Red anchors denote the segment over which the weighted linear regression was performed to determine the neutral model's indel rate parameter ρ (central blue curve). An overrepresentation of long ungapped segments is apparent in all whole-genome histograms, and especially for higher G+C contents. In contrast, the histograms that include only AR data show a tight fit to the neutral model, with only modest overrepresentation of long segments.

**Figure 3:**
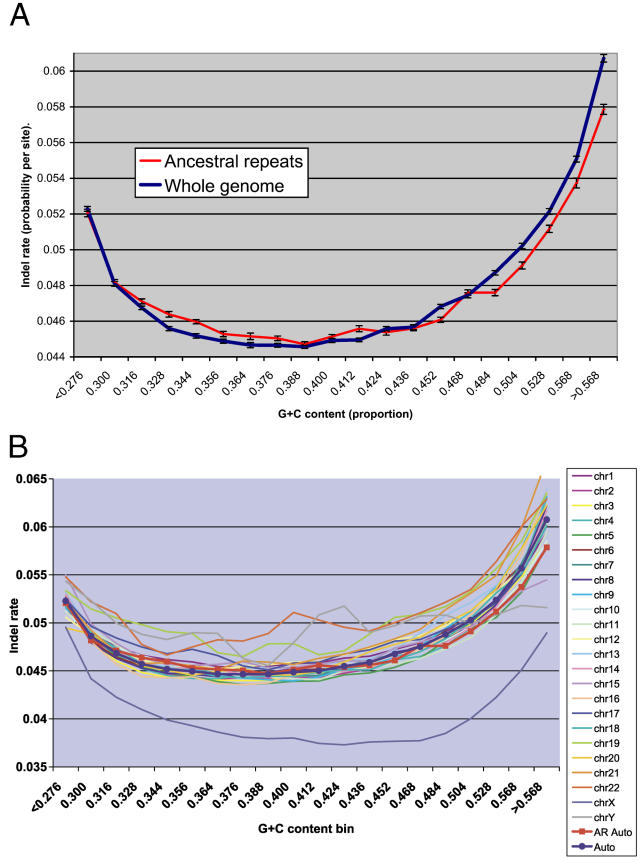
Indel Rate Variation by G+C Content (A) Whole genome (blue) and AR (red) averages of indel rates. Error bars denote 95% confidence intervals in ρ as determined by weighted linear regression on log frequencies in the intergap length histogram. (B) Indel rates per G+C content for individual chromosomes (error bars not included for clarity), and autosomal averages (whole autosome, blue; ARs, red). Most autosomes have undergone similar indel rates, with mildly increased rates for the small chromosomes (22 and 19 in particular), and a marked reduction for X, as expected by its distinct germline history. Because of its size, measurements on the Y chromosome lack accuracy, but are consistent with an increase in indel rates.

To partially account for this, we divided the human genome into 20 bins on the basis of G+C content within 250-bp windows, adjusting thresholds to make bins contain equal fractions of the genome, and IGS-length histograms were generated for each bin (Figure 2). Indel rates vary substantially for different G+C fractions (ρ = 0.0446–0.0607, Figure 3A), with the highest rates for the extreme G+C–rich and G+C–poor ends of the scale. Computing the weighted average of all *σ* values obtained from the histograms, we found *σ* = 0.1142 for the whole genome, and *σ* = 0.0087 for AR data.

A second expected source of indel rate variation is germline history, which is different for sex chromosomes and autosomes. To test this, indel rates were measured for each chromosome separately (Figure 3B). The autosomes are under broadly similar indel rates, both on average and as a function of G+C content, with the exception of Chromosomes 19 and 22, where indel rates are moderately but significantly higher (*z*-score 18 and 11, respectively). The largest outlier is the X chromosome which exhibits an indel rate (0.0400) that is 15% lower than the autosomal average (0.0480). The small size of the Y chromosome reduces the accuracy of the rate measurements, but results are consistent with a moderately increased rate compared to that of autosomes. Accounting for the lower indel rate on the X chromosome by constructing histograms for the X and for the rest of the genome separately, each binned by G+C content as before, resulted in weighted average *σ* values of 0.1131 (whole genome) and 0.0069 (ARs).

Accounting for indel rate variation as described above thus reduces overall *σ* values only marginally. Because of the relatively large extent of the observed indel rate variation, it is perhaps surprising that the original whole-genome histogram for ARs (Figure 1B) exhibits so little convexity. To investigate this, IGS lengths were simulated under the neutral model according to the G+C–dependent indel rates observed on autosomal and X-chromosomal AR data separately, and combined into a single histogram. This simulated histogram displayed little convexity and a tight fit to a single geometric, with *σ* = 0.0002 (Figure S1). The *σ* measure thus appears to be relatively insensitive to variations in neutral indel rates on the scale observed.

Investigating the AR histogram more closely, we observed a number of remarkably long ungapped segments, with 25 of these longer than 500 bp. All 25 align to mouse fragment sequence that is unplaced within assembled mouse chromosomes and that shows extraordinarily few (<1%) substitutions compared with human, whereas the corresponding dog, rat, and chimpanzee sequences exhibit gap and substitution patterns that are consistent with neutral evolution. It thus appears likely that these segments represent contaminants, and are primate, not mouse, sequence. A whole-genome scan identified 146 fragments exhibiting similar characteristics, contributing 285 kb to the human–mouse alignment (0.03%, Table S1). Removing these fragments reduced the *σ* value for ARs to 0.0067, while increasing the whole-genome *σ* to 0.1134.

**Figure 4:**
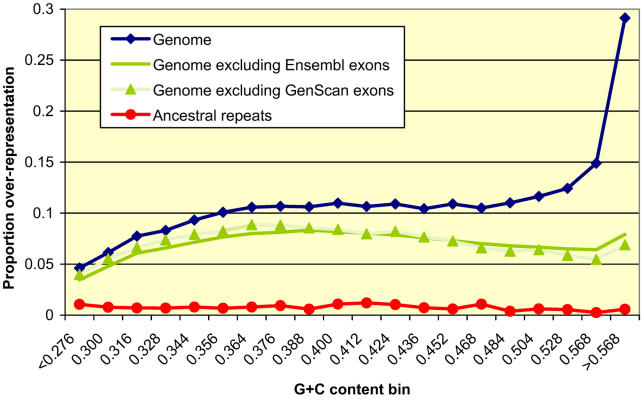
Extent of Indel-Purifying Selection in the Human Genome by G+C Content Vertical axis shows *σ* (fraction of nucleotides in ungapped segments that are overrepresented with respect to predictions of the neutral indel model) in human–mouse alignments, for the whole genome (blue), whole genome without exons (green, Ensembl exons including UTRs; shaded green, GenScan exons), both relative to 1,002-Mb mouse-aligning bases, and overrepresentation relative to 177 Mb of ARs (red). In all cases, overrepresentation on the X chromosome was measured separately; values shown are for all chromosomes combined. The measured overrepresentation of long ungapped segments is mainly due to indel-purifying selection, and in part to neutral indel rate variation and other causes (see the section Accounting for Indel Rate Variation). The exclusion of annotated exons, which tend to reside in G+C–rich regions of the genome, all but removed the peak at the highest G+C quantiles, indicating that non-genic functional material tends to accumulate at intermediate G+C levels.

The stratification of the genome by G+C content allowed the distribution of material under indel-purifying selection by G+C content to be investigated, using *σ* as an indicator for the amount of such material (Figure 4). This distribution shows a marked peak at the highest G+C quantiles. To investigate to what extent this is caused by conserved protein-coding exons and their preference for G+C rich regions [1], and the high G+C content of exons themselves, this analysis was repeated excluding all segments overlapping Ensembl- and GenScan-annotated exons. The remaining distribution is largely uniform, apart from a small shoulder for the highest quantile possibly caused by unannotated exons, or recent pseudogenes that have not yet accumulated many indels. The clearly distinct distributions of exonic and non-exonic conserved sequences with respect to G+C are consistent with previous observations that the majority of conserved material is non-protein-coding [17].

### Bounds on Selection

The results above imply that less than 1.2 Mb of human DNA annotated as TEs is unaccounted for by the neutral model (a proportion 0.0067 of 177 Mb human–mouse ARs, or less than 0.09 % of all human TEs). A fraction of this will be due to residual indel rate variation which has not been accounted for in the analysis. Other contributions may include non-orthologous alignments, which are more prevalent for repetitive sequence, and misannotations. Finally, a fraction of the 1.2 Mb TEs may be truly under indel-purifying selection, and thus have (or have had) a functional role in human biology.

**Figure 5:**
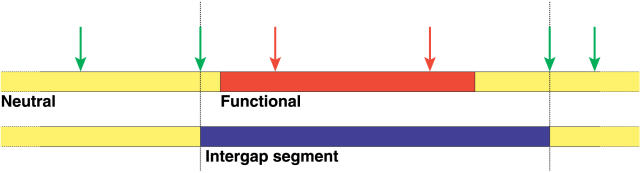
Model for the Relation between Ungapped and Functional Segments Indel events (modeled as point events, and represented by arrows) affecting functional DNA (red) are purified from the population and are not observed in extant species. The remaining indels (green arrows) delineate ungapped segments. Those subtending a segment of functional DNA (dark blue) are longer than the functional element itself, and the amount of neutral sites included in these long ungapped segments is on average twice the expected distance between indels on neutrally evolving DNA (see Materials and Methods).

Bounds on the proportion of material under indel-purifying selection in the human genome were then derived. To do this, we had to account for the fact that not all DNA in long ungapped segments is expected to be under selection owing to the relatively low density of indels. As a simple model, the genome was considered to consist of segments of functional material that is purified of any indel, separated by neutral material that accepts all indels (Figure 5), and indels were further simplified as point events. Under this model, functional segments are contained within ungapped segments that include a proportion of neutral material (neutral overhang) at both ends of the segment. For isolated functional segments, the average amount of neutral sites included in this way is *2K,* where *K = ρ^−1^* is the neutral expected distance between indels. For densely clustered functional segments, the average contribution of neutral sites drops because they are shared between segments. It can be shown that for such regions, the expected number of neutral sites per segment is *K* (see Materials and Methods).

To derive the upper bound on the proportion of indel-purified human DNA, it was assumed that only purifying selection contributes to the observed whole-genome *σ* value of 0.1134, with no contribution of residual rate variation, and high clustering was assumed which implies a low average neutral overhang of *K* nucleotides per ungapped segment. Combining data from all histograms under these assumptions, an upper bound of 100.0 ± 0.8 Mb, or 3.25% of human euchromatin, was found.

For the lower bound, the observed *σ* on ARs (0.0067) was used as an upper bound for the contribution of residual rate variation to the whole-genome *σ* value. Effectively, this assumes that all AR-annotated elements are evolving neutrally, and that no misalignments have inflated the AR estimate of *σ*. Consequently, the derived lower bound is considered a conservative estimate. Further, low clustering of functional segments was assumed, thereby implying an average neutral overhang of *2K* sites per segment. These assumptions led to a lower bound of 78.8 ± 0.6 Mb, or 2.56% of human euchromatin.

### Identification of Sequence under Selection

The resolution for identifying DNA under indel-purifying selection is limited by the relatively low human–mouse indel rate of one per 16–22 surviving homologous sites. To improve resolution, the dataset was augmented by the dog genome. This choice was motivated by the high quality of the dog assembly [18], and because, being an outgroup to human and mouse, the dog genome adds considerable resolution, increasing the effective autosomal indel rate to 0.0624–0.0909 (depending on G+C content), or one per 11–16 sites. The methodology and justification of the model and method remain unchanged, with ungapped segments now referring to segments in three-way alignment blocks devoid of gaps in all of the three species. Apart from the higher overall indel rate resulting in a steeper slope, the IGS-length histograms have a similar shape to those for human–mouse (Figure S2).

**Figure 6:**
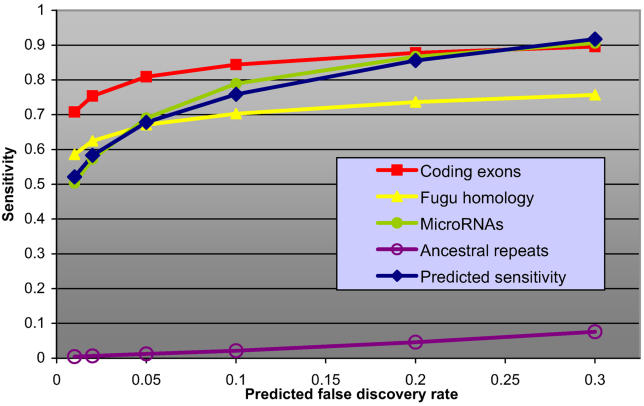
Experimental and Predicted Sensitivity versus Predicted FDR Axes show predicted proportion of neutral nucleotides (horizontal) and proportion of identified nucleotides among mouse-aligning nucleotides within annotation class (vertical). Red, yellow, and green curves show partial sensitivity to (known or likely) functional DNA, with the predicted sensitivity to DNA under indel-purifying selection (blue curve) following their general trend. For a fair comparison, the partial sensitivities were computed relative to the material common to human, mouse, and dog. The purple curve charts the sensitivity for neutrally evolving ARs, for comparison. Note that the false positive fraction (relative to mouse-aligning neutral elements) is considerably lower than the predicted FDR (relative to the identified set). Converting to the false positive fraction, we calculate the area under the resulting receiver-operating-characteristic curve to be high at 0.93, indicative of the method's discriminatory power.

We identified a set of segments highly enriched with indel-purified DNA by setting thresholds on the length of ungapped segments. The neutral indel model was used to predict the number of segments expected to exceed any length threshold under neutrality, from which we calculated the FDR. Adjusting for neutral overhang and false positives, we computed the amount of material under indel-purifying selection among the identified segments, and from this the sensitivity was estimated (see Materials and Methods). This prediction was compared with the method's observed sensitivity to identify known functional material, such as coding exons and microRNAs, and to sequence exhibiting deep conservation which is highly likely to represent functional material [19]. The predicted sensitivity follows the general trend shown by all three partial sensitivities (Figure 6), indicating that the prediction is accurate.

At a 1% FDR, we obtained 54.44 Mb of human DNA that is refractory to indels, which includes 64.0% (23.44/36.61) Mb of known coding exons [20]. This set also includes 48.7% (9,272/19,039 bp) of 222 currently annotated microRNAs [21], which is remarkable considering that these elements are dispersed among the 860 Mb of human DNA that aligns with mouse and dog. Allowing a 10% FDR, these proportions rise considerably, to 76.4% (27.96/36.61 Mb) for protein-coding exons, and 76.3% (14,524/19,039 bp) for microRNAs, within a set of 89.67 Mb of identified segments. Of 36 additional, newly discovered human microRNAs that show conservation beyond primates, which were published after this study was completed [22], 25 were found to share overlap with indel-conserved segments at this FDR level.

Besides these known elements, the majority of the segments identified consists of currently unannotated sequence (66.6% at 10% FDR), which we predict to predominantly represent DNA that has been under indel-purifying selection. This prediction, implied by the predicted FDR and sensitivity, is supported by the distribution of the identified elements with respect to various annotations (Table 1). Two annotations in particular stand out. The density of TEs (3.3%, 2.94/89.67 Mb) within the set of identified segments is more than 10-fold lower than the whole-genome density (41.1%, 1,263/3,077 Mb), while the density of DNA exhibiting homology to chicken or *Fugu* (52.7%, 47.27/89.67 Mb) is more than 10-fold higher than the overall density in the human genome (4.0%, 123/3,077 Mb).

**Table 1:**
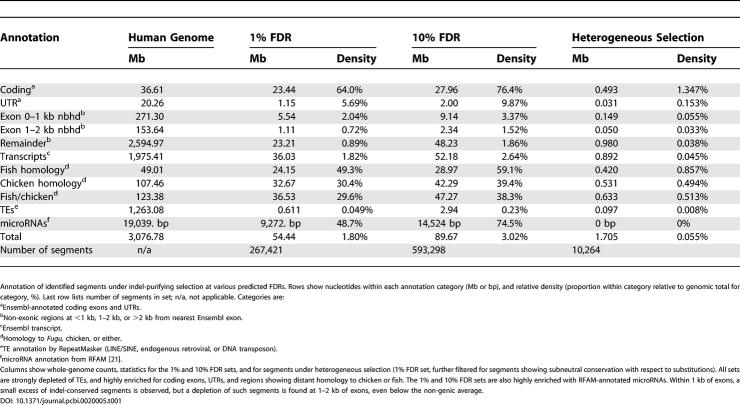
Annotation of Sequence under Indel-Purifying Selection

**Figure 7:**
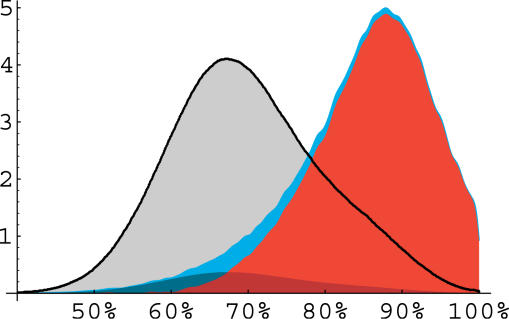
Empirical Distribution of Sequence PID to Mouse Shown is the PID distribution for human segments under indel-purifying selection (at a 1% FDR; blue), and a background distribution obtained on putatively neutrally evolving segments (non-exonic, and not in identified set of segments at 10% FDR; grey). The blue distribution can be decomposed as a mixture of 6% background (shaded) and a remainder (red), suggesting that a proportion of ungapped elements (≈ 5%, mixture coefficient minus FDR of 1%) are under purifying selection with respect to indels, while evolving under relaxed constraints or positive selection with respect to substitutions (see Materials and Methods).

To investigate the relationship between purifying selection with respect to either substitutions or indels, we computed the empirical distribution of percent nucleotide identity (PID) of aligned human and mouse sequence, using the segments identified at the 1% FDR level (Figure 7). While indel-purified DNA is generally highly conserved with respect to substitutions, as expected (mode of distribution at 88% PID), it is notable that the distribution of PIDs has a marked tail towards low values. Although in part this can be explained by the 1% false positives, it was found that the tail accounts for 6% of the total distribution (see Materials and Methods), which suggests that the identified segments include a proportion of DNA under heterogeneous selection. To support this claim, the 1% FDR set was filtered for segments exhibiting less than the neutral mean PID, as determined for each G+C content bin separately (neutral mean PID ranging from 68.2% to 65.7%, averaging 67.1%). The resulting set of 1.705 Mb still showed strong enrichment with coding exons (29%, 0.493/1.705 Mb) and with DNA exhibiting ancient ancestry (*Fugu;* 25%, 0.419/1.705 Mb), and a dearth of TEs (5.7%, 0.097/1.705 Mb) compared with the whole-genome densities (coding exons, 3.8%; *Fugu-*homology, 4.8%; TEs, 16%; all figures are relative to mouse- and dog-aligning DNA). This suggests that despite the lack of substitution-based conservation, this set still contains a considerable amount of functional material.

## Discussion

We have introduced a simple model for the neutral genomic distribution of indels, predicting a geometric drop-off in the frequency of intergap distances across whole-genome alignments. This prediction was observed to hold nearly exactly in human–mouse alignments across a range of intergap distances (20–50 bp). By realigning human Chromosome 21 sequence, this effect was shown to be independent of the alignment procedure, and appears instead to reflect the signature of past mutation. The distribution of between-indel distances within human ARs was shown to agree closely with the neutral model predictions (no statistically significant deviations across the range 20–80 bp), and the total amount of ARs under sustained purifying selection with respect to indels was shown to be at most 1.2 Mb, or 0.09% of all TEs.

A few examples of co-opted and functional TEs are known [23,24], and additional TEs that show high conservation have been found [7]. Further examples found in this study include a Charlie10/MER1 DNA transposon in the last intron of the zinc finger gene *ZNF258,* exhibiting no indels over a 1,322-bp region, and an L3/CR1 LINE element in the penultimate intron of the putative gene *C10orf11*. Despite these examples, the estimated fraction of 1.2 Mb of ARs under purifying selection is probably a large overestimate, and more detailed studies are needed to answer to what extent TEs functionally contribute to human biology. Because of their large copy number, it is possible that TEs have functional (symbiotic) roles despite also evolving neutrally after integration into the genome [25,15]. However, our results do demonstrate that the vast majority of human ARs (>99.3%) have not been under sustained purifying selection. This is significant since, although ARs commonly have been assumed to have evolved neutrally, this had not previously been quantified using a neutral model.

Substantial indel rate variation with local (250-bp) G+C content was observed, with up to 35% increased indel rates for both high and low G+C content. This observation is consistent with polymerase slippage as a main cause of indels, since extremes in G+C content imply higher expected sequence similarities, thereby facilitating slippage. Indel rates also vary with chromosome type, with X having a 15% lower average indel rate than the autosomes. For substitutions, a similar pattern has been observed [26]. This is likely due to X spending two-thirds of its evolutionary history in females, and undergoing fewer mutations than autosomes which dwell equally in either sex.

In contrast to ARs, the distribution of inter-indel distances for the whole genome significantly departs from the neutral model, exhibiting a large excess of long (>>50-bp) ungapped segments. Because purifying selection is expected to result in long ungapped segments, and because at most a vanishing fraction of ARs is believed to have been under purifying selection, in stark contrast to the rest of the human genome, these observations are consistent with purifying selection being the predominant cause for this departure. Variations in indel rates that were not accounted for in principle also will cause deviations of this qualitative type. However, ARs are ubiquitous in the human genome, and large-scale rate variations would also influence the distribution of indels within ARs. Local rate variations due to sequence features specific to AR (or non-AR) DNA may also give rise to residual rate variations, and indeed small differences in indel rates were found between ARs and general genomic DNA, after accounting for G+C content. These differences may have various causes, including differences of G+C content distribution within bin thresholds and indel rate variations due to differences in sequence composition other than G+C content. However, simulations showed that rate variations as large as 35% cause a departure from the neutral model of almost three orders of magnitude less than that observed for the whole genome (*σ* = 0.0002 versus *σ* = 0.1134, Figure S1C), so that such residual rate variations are unlikely to account for the observations.

Using several conservative assumptions, the total amount of DNA under indel-purifying selection was estimated to be between 2.56% and 3.25% of human euchromatin. These estimates are lower than an earlier estimate of 5% based on an analysis of nucleotide substitutions between human and mouse [1,2]. Several factors may contribute to this difference, including a degree of independence between substitution-based and indel-based selection, unaccounted-for genomic substitution-rate variations, and alignment errors, in particular non-orthologous alignments between repetitive regions. It also has to be noted that the substitution-based study aimed to estimate the proportion of 50-bp windows containing evolutionary conserved sequence, and so included an unknown proportion of nucleotides not under selection, while the indel-based estimate accounts for such neutral contributions. It must also be emphasized that our observations have taken advantage of ancient evolutionary events and thus cannot account for sequence which has recently gained or lost selective constraint. Functional elements that have evolved recently, that were lost in mouse, or that have been evolving under relaxed constraints or under positive selection with respect to indels, all will not be included in the current estimate. Conversely, elements that have recently lost their function but have not yet accumulated many indels, such as certain classes of recent pseudogenes, will be unjustly included. Finally, sequence elements that have phenotypic relevance but whose selection coefficient is too small in relation to the effective population size, will not have been effectively purified of deleterious mutations, rendering such regions unobservable in comparative analyses.

We identified a set of ungapped segments highly enriched with material under indel-purifying selection, and containing a small and predetermined fraction of neutrally evolving segments (FDR). This set was found to be highly enriched with coding exons, known microRNAs, and DNA-sharing homology with chicken and *Fugu,* while strongly depleted of ARs. This provides a direct and independent confirmation for the interpretation that the overrepresentation of long ungapped segments is due to the purification of deleterious indels. Using the bounds on the total amount of material under indel-purifying selection discussed above, estimates for the sensitivity of the method were obtained as 52% and 76% for FDRs of 1% and 10%, respectively. The observed sensitivities for identifying known exons and *Fugu-*aligning DNA broadly agree with this estimate. Indeed the sensitivity for known microRNAs almost exactly tracks the predicted sensitivity curve, while the observed sensitivity for coding exons is higher than predicted, possibly because of the strong deleterious effect of frame-changing indels.

While most of the material identified is unannotated, the low overall density of ARs, the high average PID to mouse, and the high density of chicken- and *Fugu-*aligning material all suggest that a large majority of identified sequence represents functional material that has been under purifying selection with respect to both indels and substitutions. It thus appears that the identified material predominantly represents sequence that has been, or remains, functional in mouse and/or human lineages.

The method's sensitivity to microRNAs and protein-coding exons is remarkable for a method that uses neither structural nor evolutionary models particular to exon or microRNA sequence, nor any substitution-based conservation approach, and suggests that the present method will be advantageous as part of a computational gene or microRNA discovery tool. The simplicity of the proposed method easily allows other signals to be included, too. For example, functional material is expected to be highly clustered, and indeed a high degree of clustering was found among the segments we identified (50% of identified segments are within 250 bp of another, and 20% are within 10 bp; expected proportions for a uniform distribution are 4% and 0.2%, respectively). One way of exploiting this would be to consider consecutive indels, and derive a neutral model for such configurations. Finally, although indel spectra vary considerably between organisms [27], the limited number of assumptions made in the model suggests that it could be fruitfully applied to other organisms, such as *Drosophila* species whose genomes are now becoming available.

Analyzing the pattern of substitutions among the identified elements, an unexpectedly large fraction was found whose conservation with respect to substitutions was indistinguishable from neutrality (about 6%, within a set predicted to contain 1% false positives). This result could be explained if, by a failure of the model, the false positive rate was grossly underestimated. However, considering only those elements exhibiting subneutral conservation with respect to substitutions, it was found that the resulting set, although naturally enriched with false positives, still contained an appreciable fraction of functional elements, as indicated by a strong depletion of ARs, and an enrichment with coding exons and *Fugu-*aligning material. We hypothesize that a proportion of these elements represents elements that have been under heterogeneous selection, i.e., under indel-purifying selection, but under positive selection or relaxed constraint with respect to nucleotide substitutions. This is an exciting possibility since, lacking a non-comparative method for identifying functional elements other than protein-coding genes, large-scale computational identification of non-genic functional elements have hitherto relied on substitution-based comparative methods, which prohibits the identification of elements under positive selection with respect to substitutions. Elements under heterogeneous selection form a subset of these elements, and the present method's ability to identify such elements enables the investigation of positive selection on non-genic functional elements. Non-genic conserved elements have been implicated in developmental pathways [5,6], which are prime targets for adaptive evolution, and the ability to identify non-genic elements that are evolving adaptively opens new avenues into the investigation of the relationship between these functional elements and phenotype.

The full data set of identified segments, at both 1% and 10% FDRs, is available for download and visualisation in genome browsers at http://www.stats.ox.ac.uk/~lunter/IGS.

## Materials and Methods

### Sequences and annotations.

We installed mirrors of four sets of BlastZ alignments [13] from UCSC Genome Bioinformatics, http://genome.ucsc.edu (Santa Cruz, California, United States): human/mouse (hg17vsMm5), the 8-way human-based alignment (multiz8way) of which we retained only the human, mouse, and dog tracks (assemblies: human, NCBI build 35; mouse, NCBI build 33; dog, MIT Broad Institute July 2004 build), human/*Fugu* (hg17Fr1), and human/chicken (hg17vsGalgal2). To remove a minority of possible spurious alignments caused by repetitive sequence, we filtered for conserved synteny by keeping alignment chains only when proximal to trusted anchors (within 100 kb on the human assembly; within 1 Mb on the mouse assembly), removing 2.43 Mb of alignments (human/mouse). Trusted anchors were defined as alignable segments exceeding 150 bp without RepeatMasker [28] or TandemRepeatFinder [29] annotation. Putative primate contaminants of mouse sequence were identified by screening for ungapped human–mouse alignments more than 200 bp in length exhibiting >99% sequence identity, with no supporting evidence for high conservation (defined as >80% identity, less than one gap per 50 bp) in either rat or dog with respect to the human sequence. Protein-coding gene annotations were taken from Ensembl [20]; annotations for microRNA genes were taken from RFAM [21].

### Intergap length histograms.

IGS were defined as aligned segments of homology, uninterrupted by gaps in any of the two alignment tracks (or three in case of human–mouse–dog alignments). The neutral model was fitted to the observed histogram counts by weighted linear regression on the log frequencies, with weights derived from the expected sampling error per length bin (binomial distribution) in log-space. The length intervals over which this regression was performed were determined by maximizing the coefficient of determination *(R^2^),* typically reaching 0.9996–0.9998 for G+C–binned autosomal data. The parameter *σ* was determined by counting the nucleotides represented in the histogram, and subtracting those expected from the neutral model, ignoring the leftmost (<20 bp) part of the histogram where counts are distorted by the effects of gap attraction; i.e., 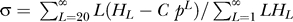 *,* where *H_L_* is the histogram count for length *L,* and *C* is the scaling constant of the neutral model.

Histograms on the AR portion of the genome were obtained by intersecting IGS with segments annotated as AR by RepeatMasker [28]. The intersection procedure causes a bias towards short IGS because of the finite extent of ARs. To account for this, we first estimated the indel rate based on the raw histogram, and then corrected for the premature cutoff by convoluting the length histogram for IGS that overlapped the right end of an AR with the geometric length distribution obtained from the indel rate estimate. This adjustment, which is equivalent to stochastically extending those IGS according to the neutral model, is both modest (Figure S1) and conservative, since the resulting *σ* value for the adjusted histogram exhibits only a slight increase, which has a conservative effect on all estimates based on it.

### Realignment of Chromosome 21.

Realignment was performed using a probabilistic aligner, implementing a pair hidden Markov model (p. 82 of [30]). We first estimated the indel rate at ρ = 0.0491 ± 0.0003 (one standard error) from BlastZ data. Realignment was performed on the alignment fragments as found by BlastZ of length at most 5,000 bp (representing >99% of nucleotides), after removing all gaps. Two sets of indel parameters were used, δ = 0.025 representing the best-known indel rate (ρ = 2δ, see [30]), and δ = 0.030. Other parameters were fixed at ɛ = 0.667 (the indel length distribution parameter) and τ = 0.001. This 20% change in the alignment indel rate parameter resulted in a statistically non-significant 1% change in the observed ρ (0.0484 ± 0.0004 and 0.0491 ± 0.0004, respectively), with both values within 95% confidence limits of the value determined from BlastZ [13] data. We next replaced the substitution probability matrix (obtained by a Baum-Welch training procedure) by a Kimura 2-parameter model which assumes equilibrium probability 1/4 for all nucleotides (parameters a = 0.18; b = 0.07). For these parameter values, the mean absolute deviation to the matrix coefficients to the original joint probability matrix was 18.5%, while the observed ρ changed by less than 0.5% (ρ = 0.0482 ± 0.0004).

### Contribution of neutral sites to long IGS.

The expected distance to the nearest indel downstream (or upstream) of any site is *K = ρ^−1^*, the expected distance between indels (see Results for the definition of the conditional probability *ρ = 1−p*). In particular, the distance to the nearest indel from either end of an isolated functional element is *K,* so that the expected contribution of neutral sites to the ungapped segment containing the functional element is *2K*. Conversely, consider a region of DNA of length *T>>K* dense with functional segments, from which an unknown proportion *q* of indels has been purified, and suppose this region consists of *N* ungapped segments of average length *L > K*. The total expected number of indel events is *T/K,* so that the expected observed number is *n = (1−q) T/K* and so *L = T/n = K (1−q)^−1^* in expectation. The expected amount of DNA under indel-purifying selection is thus *q T = q N L = (1 − K/L) N L = N (L−K)*, i.e., each of the *N* ungapped segments contains on average *K* neutral sites. Therefore, under this model, the expected amount of neutral sites within ungapped segments containing functional material is between *K* and *2K,* depending on the extent of clustering.

### Identifying indel-purified segments.

For a given FDR (defined as the predicted proportion of neutral segments among those identified, weighted by sequence length), we obtain thresholds on ungapped segment length for all G+C bins, and for the X chromosome and other chromosomes separately, by constrained maximization of the predicted total amount of identified segments under selection, using the method of Lagrange multipliers. The predicted sensitivity was computed by adjusting for the contribution of neutral sites using the upper bound method described, and dividing by the whole-genome upper bound of 100 Mb.

### Analysis of PID.

We computed the histogram of Figure 7 by calculating the PID as *100 ( I+ɛ ) / L* (*I,* the number of identical nucleotides between human and mouse; *L,* the segment length; *ɛ* uniformly drawn from [0,1] for histogram smoothing), then adding *L* counts to the histogram bin to weight by the number of nucleotides, using a bin size of 0.1%. A PID histogram was constructed both for segments under purifying selection, and for a subset of segments deemed to have evolved neutrally (defined as not overlapping an exon, and not being among segments under indel-purifying selection at the 10% FDR level). To ensure that the variances in the two histograms were comparable, we matched segment-length distributions by sampling lengths for segments under indel-purifying selection according to the observed neutral-segment–length distribution, centering the subsegment within the original segment. We required segments for either histogram to be at least 40 bp long. To obtain the proportion of nucleotides under heterogeneous selection, we considered the first histogram to be a mixture of the neutral distribution (second histogram) and an unknown distribution (Figure 7). Subtracting the largest possible proportion of neutral distribution, requiring the difference to remain strictly positive, we obtained a mixture coefficient of 0.06.

## Supporting Information

Figure S1 Influence of Stochastic Extension and Geometric Mixture on AR-IGS Histograms Horizontal axis, IGS length; vertical axis, log_10_ of histogram counts. Blue curves, weighted linear regression fit (centre curve; interval 16–77), and 95% confidence limits for the histogram counts under the model (outer curves).
(A) Histogram of IGS truncated to AR boundaries (*σ* = 0.0061)
(B) Same histogram after stochastic extension of truncated IGS overlapping the rightmost end of ARs (see Materials and Methods) (*σ* = 0.0074). Stochastic extension of truncated IGS has a minor influence on the final histogram and results in a modest increase in the *σ* value. This adjustment is conservative for all bounds that depend on this value (upper bound on ARs, and lower bound on general genomic DNA under purifying selection). (C) Histogram of simulated IGS lengths obtained by mixing lengths drawn from 40 geometric distributions (20 G+C content values, and X/non-X), with indel rates and number of segments as for human–mouse ARs (*σ* = 0.0020). The resulting mixture distribution remains close to a single geometric, and the distribution has a vanishingly small *σ*. We conclude that indel rate variation on the scale observed in the human genome does not by itself explain the large *σ* value observed for the whole-genome IGS histogram.
(67 KB DOC)

Figure S2 Genomic Distribution of Intergap Distances in Human–Mouse–Dog Alignments Histogram of intergap distance counts (log_10_ scale) in human–mouse–dog alignments, (A) within the whole genome and (B) within ARs. See Figure 1 caption for more details. The histograms are similar to those for human–mouse alignments, except for a steeper slope in the neutral region due to a higher combined indel rate in alignments of the three species, leading to an increased sensitivity for identification of segments under indel-purifying selection.
(236 KB DOC)

Table S1 Unplaced Mouse Sequence Fragments Showing Very High Conservation (>99% Sequence Identity, No Gaps) Relative to Human DNA, but No Supporting Evidence for Conservation in Dog or Rat These 146 fragments, of which 285 kb align to human DNA in the BlastZ alignments we used, most likely represent sequence contamination from primates and were thus subsequently excluded from our analysis.
(48 KB DOC)

## Abbreviations

AR: ancestral repeat
bp: base pairs
FDR: false discovery rate
IGS: intergap segments
PID: percent nucleotide identity
TE: transposable element
