# Supplementary material for: Genome-Wide Identification of Human Functional DNA Using a Neutral Indel Model
Source: PLoS Comput Biol. 2006 Jan 13;2(1):e5. doi: 10.1371/journal.pcbi.0020005 (PMC1326222; doi:10.1371/journal.pcbi.0020005)
Supplement: Supplementary file 1 [file pcbi.0020005.sg001.doc]

Figure S1 **a**

**b**

**c**

Influence of stochastic extension and geometric mixture on ancestral repeat (AR) IGS histograms. Horizontal axis, IGS length; vertical axis, log_10_ of histogram counts. Blue curves, weighted linear regression fit (centre curve; interval 16-77) and 95% confidence limits for the histogram counts under the model (outer curves). (**a**), histogram of IGS truncated to AR boundaries (*σ* = 0.0061), and (**b**) same histogram after stochastic extension of truncated IGS overlapping the rightmost end of ARs (see Methods) (*σ* = 0.0074). Stochastic extension of truncated IGS has a minor influence on the final histogram, and results in a modest increase in the *σ* value. This adjustment is conservative for all bounds that depend on this value (upper bound on ARs, and lower bound on general genomic DNA under purifying selection). (**c**) histogram of simulated IGS lengths obtained by mixing lengths drawn from 40 geometric distributions (20 G+C content values, and X/non-X), with indel rates and number of segments as for human-mouse ARs (*σ* = 0.0020). The resulting mixture distribution remains close to a single geometric, and the distribution has a vanishingly small *σ*. We conclude that indel rate variation on the scale observed in the human genome does not by itself explain the large *σ* value observed for the whole-genome IGS histogram.
