# Supplementary material for: Genome-Wide Identification of Human Functional DNA Using a Neutral Indel Model
Source: PLoS Comput Biol. 2006 Jan 13;2(1):e5. doi: 10.1371/journal.pcbi.0020005 (PMC1326222; doi:10.1371/journal.pcbi.0020005)
Supplement: Supplementary file 2 [file pcbi.0020005.sg002.doc]

Figure S2. Genomic distribution of intergap distances in human-mouse-dog alignments.

**a**

**b**

Histogram of intergap distance counts (log_10_ scale) in human-mouse-dog alignments, (**a**) within the whole genome and (**b**) within ancestral repeats. See the caption of Figure 1 for more details. The histograms are similar to those for human-mouse alignments, except for a steeper slope in the neutral region due to a higher combined indel rate in alignments of the three species, leading to an increased sensitivity for identification of segments under indel-purifying selection.
