# Supplementary material for: Genome-Wide Identification of Human Functional DNA Using a Neutral Indel Model
Source: PLoS Comput Biol. 2006 Jan 13;2(1):e5. doi: 10.1371/journal.pcbi.0020005 (PMC1326222; doi:10.1371/journal.pcbi.0020005)
Supplement: Supplementary file 3 [file pcbi.0020005.st001.doc]

```
Mouse chromosome         start          end             Fragment        Human chrom      start          end
mm5.chrUn_random         1670203         1671521        CAAA01075473.1  hg17.chr1       34266577        34267296
mm5.chrUn_random         2763841         2765880        CAAA01078169.1  hg17.chr15      74777870        74778147
mm5.chrUn_random         3339003         3350479        CAAA01079499.1  hg17.chr2       49208656        49209460
mm5.chrUn_random         3358645         3364338        CAAA01079523.1  hg17.chr2       49287952        49289216
mm5.chrUn_random         3365338         3368656        CAAA01079526.1  hg17.chr2       49289216        49290320
mm5.chrUn_random         3647516         3649472        CAAA01080225.1  hg17.chr18      30905184        30905830
mm5.chrUn_random         3736273         3739980        CAAA01080460.1  hg17.chr2       180614648       180618333
mm5.chrUn_random         4445299         4447357        CAAA01081827.1  hg17.chr11      122351224       122352817
mm5.chrUn_random         4611385         4612457        CAAA01082256.1  hg17.chr8       53547337        53547666
mm5.chrUn_random         4643425         4644830        CAAA01082290.1  hg17.chr9       28311752        28313056
mm5.chrUn_random         5823368         5824869        CAAA01083935.1  hg17.chr11      122394436       122395870
mm5.chrUn_random         6055196         6056701        CAAA01084365.1  hg17.chr2       77467739        77468485
mm5.chrUn_random         6080811         6085061        CAAA01084441.1  hg17.chr2       180460130       180465374
mm5.chrUn_random         6086061         6088712        CAAA01084442.1  hg17.chr8       111638493       111640963
mm5.chrUn_random         7311770         7312857        CAAA01086216.1  hg17.chr15      77779327        77780273
mm5.chrUn_random         8507248         8508543        CAAA01088626.1  hg17.chr8       136516876       136517659
mm5.chrUn_random         8554188         8554970        CAAA01088744.1  hg17.chr2       140965821       140966507
mm5.chrUn_random         8667391         8668719        CAAA01088996.1  hg17.chr2       140828468       140829317
mm5.chrUn_random         9291787         9292861        CAAA01090199.1  hg17.chr1       6488933         6489738
mm5.chrUn_random         9578190         9583814        CAAA01091129.1  hg17.chr2       140835767       140840780
mm5.chrUn_random        10013202        10017249        CAAA01092285.1  hg17.chr2       140950319       140964045
mm5.chrUn_random        10041975        10044911        CAAA01092334.1  hg17.chr2       140890076       140908145
mm5.chrUn_random        10048175        10050079        CAAA01092352.1  hg17.chr2       180469759       180470109
mm5.chrUn_random        10051079        10068357        CAAA01092365.1  hg17.chr2       180542955       180583055
mm5.chrUn_random        10069357        10075020        CAAA01092379.1  hg17.chr2       180583807       180584202
mm5.chrUn_random        10473203        10474496        CAAA01093244.1  hg17.chr15      31661966        31663057
mm5.chrUn_random        10480913        10482526        CAAA01093259.1  hg17.chr2       164196770       164197938
mm5.chrUn_random        10978341        10979827        CAAA01094452.1  hg17.chr15      77741480        77742627
mm5.chrUn_random        11081302        11083087        CAAA01094621.1  hg17.chr2       49190880        49191206
mm5.chrUn_random        11127510        11128630        CAAA01094684.1  hg17.chr2       140872350       140880258
mm5.chrUn_random        11168165        11169630        CAAA01094724.1  hg17.chr6       69173303        69180656
mm5.chrUn_random        11891328        11892563        CAAA01096184.1  hg17.chr15      77735759        77736588
mm5.chrUn_random        11897683        11900480        CAAA01096192.1  hg17.chr15      77743734        77745542
mm5.chrUn_random        11920378        11921771        CAAA01096258.1  hg17.chr9       28307075        28307389
mm5.chrUn_random        11948240        11950740        CAAA01096273.1  hg17.chr2       239809486       239811989
mm5.chrUn_random        12135235        12136968        CAAA01096625.1  hg17.chr4       157133289       157134512
mm5.chrUn_random        12145128        12146303        CAAA01096635.1  hg17.chr18      30966664        30972685
mm5.chrUn_random        12160416        12161695        CAAA01096643.1  hg17.chr4       149451496       149452085
mm5.chrUn_random        12164875        12167348        CAAA01096647.1  hg17.chr4       149454758       149455799
mm5.chrUn_random        12221052        12222413        CAAA01096811.1  hg17.chr8       136503628       136504957
mm5.chrUn_random        12275028        12276081        CAAA01096927.1  hg17.chr8       18939063        18939830
mm5.chrUn_random        12379270        12396546        CAAA01097145.1  hg17.chr2       172768925       172786102
mm5.chrUn_random        13013119        13021844        CAAA01098311.1  hg17.chr4       157114150       157130361
mm5.chrUn_random        13024864        13029899        CAAA01098336.1  hg17.chr4       157078835       157083013
mm5.chrUn_random        13041732        13044611        CAAA01098383.1  hg17.chr4       156993779       157044361
mm5.chrUn_random        13505296        13506334        CAAA01098964.1  hg17.chr15      98838776        98849131
mm5.chrUn_random        13654897        13656138        CAAA01099140.1  hg17.chr10      71316305        71317161
mm5.chrUn_random        13687381        13688308        CAAA01099164.1  hg17.chr10      71284411        71312491
mm5.chrUn_random        14133915        14134988        CAAA01099779.1  hg17.chr6       69225479        69226550
mm5.chrUn_random        14144942        14146256        CAAA01099815.1  hg17.chr6       69099545        69100837
mm5.chrUn_random        14147256        14148445        CAAA01099824.1  hg17.chr6       69180836        69182025
mm5.chrUn_random        14153736        14156067        CAAA01099849.1  hg17.chr6       69205129        69206633
mm5.chrUn_random        14157067        14158383        CAAA01099854.1  hg17.chr6       69208798        69209819
mm5.chrUn_random        14159383        14160726        CAAA01099857.1  hg17.chr5       94466691        94467571
mm5.chrUn_random        14165860        14166530        CAAA01099879.1  hg17.chr15      77761054        77761721
mm5.chrUn_random        14215520        14216564        CAAA01100015.1  hg17.chr15      77800401        77801445
mm5.chrUn_random        14891482        14893290        CAAA01101682.1  hg17.chr2       239812760       239814530
mm5.chrUn_random        14894290        14895336        CAAA01101685.1  hg17.chr2       140964646       140965170
mm5.chrUn_random        14896336        14898350        CAAA01101689.1  hg17.chr4       157093148       157094091
mm5.chrUn_random        14903537        14904877        CAAA01101694.1  hg17.chr2       180470153       180470988
mm5.chrUn_random        14969045        14970197        CAAA01101837.1  hg17.chr2       239815816       239816859
mm5.chrUn_random        15088030        15089486        CAAA01102188.1  hg17.chr8       122474494       122474714
mm5.chrUn_random        15096168        15099731        CAAA01102213.1  hg17.chr9       28331346        28334406
mm5.chrUn_random        15332578        15333824        CAAA01102691.1  hg17.chr18      30953055        30953784
mm5.chrUn_random        15826524        15828134        CAAA01103874.1  hg17.chr8       18930848        18931141
mm5.chrUn_random        15954713        15956138        CAAA01104092.1  hg17.chr18      30956260        30956952
mm5.chrUn_random        15957138        15958180        CAAA01104105.1  hg17.chr8       3276159         3277201
mm5.chrUn_random        16002700        16003766        CAAA01104171.1  hg17.chr8       75959481        75960547
mm5.chrUn_random        16329861        16331536        CAAA01105130.1  hg17.chr2       140870499       140871064
mm5.chrUn_random        16563099        16563762        CAAA01105526.1  hg17.chr4       157070551       157071214
mm5.chrUn_random        17097812        17099739        CAAA01106567.1  hg17.chr15      77754581        77754973
mm5.chrUn_random        17132271        17133983        CAAA01106658.1  hg17.chr8       122510677       122510993
mm5.chrUn_random        17216681        17221288        CAAA01107040.1  hg17.chr2       77461417        77465599
mm5.chrUn_random        17224382        17232936        CAAA01107051.1  hg17.chr2       77479675        77487684
mm5.chrUn_random        17233936        17247377        CAAA01107065.1  hg17.chr2       77501604        77514919
mm5.chrUn_random        17248377        17251795        CAAA01107069.1  hg17.chr2       77515245        77518044
mm5.chrUn_random        17252795        17257199        CAAA01107084.1  hg17.chr2       77538365        77542004
mm5.chrUn_random        17258199        17263993        CAAA01107088.1  hg17.chr2       77544416        77548804
mm5.chrUn_random        17282374        17290804        CAAA01107114.1  hg17.chr2       164071088       164088581
mm5.chrUn_random        17291804        17294223        CAAA01107122.1  hg17.chr2       164061649       164063892
mm5.chrUn_random        17320268        17322183        CAAA01107220.1  hg17.chr8       69564979        69566575
mm5.chrUn_random        17409847        17411212        CAAA01107693.1  hg17.chr8       122451536       122452582
mm5.chrUn_random        17426048        17427247        CAAA01107751.1  hg17.chr8       84090036        84127604
mm5.chrUn_random        17711662        17713197        CAAA01109058.1  hg17.chr10      71222738        71223126
mm5.chrUn_random        17716437        17717892        CAAA01109064.1  hg17.chr10      71314180        71314546
mm5.chrUn_random        17718892        17720548        CAAA01109067.1  hg17.chr15      98857418        98858362
mm5.chrUn_random        17729692        17730992        CAAA01109098.1  hg17.chr1       34201045        34202344
mm5.chrUn_random        17802317        17803812        CAAA01109355.1  hg17.chr15      77846554        77847587
mm5.chrUn_random        17843571        17844751        CAAA01109498.1  hg17.chr8       3263606         3264442
mm5.chrUn_random        17845751        17847417        CAAA01109502.1  hg17.chr8       3317279         3318673
mm5.chrUn_random        17853166        17854555        CAAA01109506.1  hg17.chr8       3277787         3278193
mm5.chrUn_random        17928946        17930311        CAAA01110167.1  hg17.chr8       84144958        84145851
mm5.chrUn_random        17964438        17965521        CAAA01110269.1  hg17.chr18      30992677        30993223
mm5.chrUn_random        17997944        17999047        CAAA01110550.1  hg17.chr11      32489606        32490623
mm5.chrUn_random        18006707        18007710        CAAA01110564.1  hg17.chr18      27259540        27260105
mm5.chrUn_random        18008710        18010242        CAAA01110570.1  hg17.chr11      43822650        43871518
mm5.chrUn_random        18013318        18014486        CAAA01110584.1  hg17.chr11      43771205        43771799
mm5.chrUn_random        18015486        18016552        CAAA01110587.1  hg17.chr11      60316807        60317867
mm5.chrUn_random        18017552        18018617        CAAA01110593.1  hg17.chr8       84088306        84089179
mm5.chrUn_random        18019617        18020780        CAAA01110596.1  hg17.chr15      31685608        31686517
mm5.chrUn_random        18024361        18025821        CAAA01110602.1  hg17.chr8       18934950        18936409
mm5.chrUn_random        18029311        18030994        CAAA01110608.1  hg17.chr8       18926544        18926782
mm5.chrUn_random        18031994        18033016        CAAA01110611.1  hg17.chr8       75930742        75931470
mm5.chrUn_random        18034016        18035138        CAAA01110617.1  hg17.chr8       69580970        69581308
mm5.chrUn_random        18036138        18037637        CAAA01110620.1  hg17.chr8       69696914        69698204
mm5.chrUn_random        18043774        18045549        CAAA01110631.1  hg17.chr8       69638081        69639694
mm5.chrUn_random        18051114        18052946        CAAA01110644.1  hg17.chr8       119117435       119118944
mm5.chrUn_random        18119918        18120936        CAAA01110814.1  hg17.chr8       439893          440706
mm5.chrUn_random        18136413        18137438        CAAA01110854.1  hg17.chr9       28193521        28194467
mm5.chrUn_random        18155128        18156753        CAAA01110903.1  hg17.chr15      74763891        74765372
mm5.chrUn_random        18486136        18487211        CAAA01111914.1  hg17.chr2       140919766       140920543
mm5.chrUn_random        18866977        18868127        CAAA01113011.1  hg17.chr5       131840590       131841303
mm5.chrUn_random        21085169        21086767        CAAA01119734.1  hg17.chr2       140820006       140821421
mm5.chrUn_random        21087767        21090426        CAAA01119737.1  hg17.chr2       140826339       140827869
mm5.chrUn_random        21091426        21094642        CAAA01119744.1  hg17.chr2       140832334       140835544
mm5.chrUn_random        21103057        21105069        CAAA01119754.1  hg17.chr2       140857578       140859014
mm5.chrUn_random        21106069        21109177        CAAA01119757.1  hg17.chr2       140862040       140864551
mm5.chrUn_random        21175647        21179874        CAAA01120030.1  hg17.chr2       172786758       172908609
mm5.chrUn_random        22933370        22934345        CAAA01124587.1  hg17.chr2       164095854       164120069
mm5.chrUn_random        22938288        22940553        CAAA01124602.1  hg17.chr2       164120968       164123167
mm5.chrUn_random        22941553        22944399        CAAA01124606.1  hg17.chr2       164124644       164142118
mm5.chrUn_random        24796896        24798712        CAAA01128323.1  hg17.chr11      122402655       122403777
mm5.chrUn_random        27797509        27798687        CAAA01134454.1  hg17.chr15      77707081        77716730
mm5.chrUn_random        27980704        27982060        CAAA01134815.1  hg17.chr5       131939061       131942650
mm5.chrUn_random        28850987        28852137        CAAA01136739.1  hg17.chr1       34214927        34219028
mm5.chrUn_random        29343480        29345103        CAAA01137643.1  hg17.chr2       140789963       140791587
mm5.chrUn_random        29350283        29354677        CAAA01137657.1  hg17.chr2       140813478       140817370
mm5.chrUn_random        29355677        29357667        CAAA01137661.1  hg17.chr2       140818007       140819997
mm5.chrUn_random        31202685        31204690        CAAA01141638.1  hg17.chr6       69076347        69077744
mm5.chrUn_random        33237744        33239396        CAAA01145562.1  hg17.chr6       69155325        69155800
mm5.chrUn_random        33652153        33653422        CAAA01146442.1  hg17.chr8       136520421       136520730
mm5.chrUn_random        34969181        34970248        CAAA01149121.1  hg17.chr8       122447785       122448743
mm5.chrUn_random        35076982        35078054        CAAA01149411.1  hg17.chr2       239808432       239808960
mm5.chrUn_random        36260552        36263544        CAAA01151570.1  hg17.chr2       239751598       239778505
mm5.chrUn_random        36266656        36267714        CAAA01151586.1  hg17.chr1       34338794        34343719
mm5.chrUn_random        36578026        36579583        CAAA01152530.1  hg17.chr2       239778681       239808311
mm5.chrUn_random        38262423        38263446        CAAA01156149.1  hg17.chr1       34302972        34310551
mm5.chrUn_random        39777935        39779874        CAAA01159306.1  hg17.chr2       239737750       239739031
mm5.chrUn_random        40129652        40130727        CAAA01159985.1  hg17.chr4       149535422       149535789
mm5.chrUn_random        40513513        40514587        CAAA01160955.1  hg17.chr6       30399902        30400433
mm5.chrUn_random        42616695        42618277        CAAA01164797.1  hg17.chr6       69086075        69087203
mm5.chrUn_random        45473777        45475816        CAAA01170683.1  hg17.chr11      122331933       122341237
mm5.chrUn_random        45476816        45477998        CAAA01170691.1  hg17.chr18      51013445        51014463
mm5.chrUn_random        46044380        46045751        CAAA01171800.1  hg17.chr6       69254393        69255764
mm5.chrUn_random        46321530        46322815        CAAA01172519.1  hg17.chr10      71225871        71226695
mm5.chrUn_random        46478711        46480543        CAAA01172953.1  hg17.chr10      71198733        71200091
mm5.chrUn_random        64474045        64476116        CAAA01222248.1  hg17.chr2       239743191       239745379
```

Supplementary Table 1. Unplaced mouse sequence fragments showing very high conservation (>99% sequence identity, no gaps) relative to human DNA, but no supporting evidence for conservation in dog or rat. These 146 fragments, of which 285 kb aligns to human DNA in the BlastZ alignments we used, most likely represent sequence contamination from primates and were thus subsequently excluded from our analysis.
